# ^18^F-FDG dose reduction using deep learning-based PET reconstruction

**DOI:** 10.1186/s13550-025-01269-9

**Published:** 2025-07-01

**Authors:** Ryuji Akita, Komei Takauchi, Mana Ishibashi, Shota Kondo, Shogo Ono, Kazushi Yokomachi, Yusuke Ochi, Masao Kiguchi, Hidenori Mitani, Yuko Nakamura, Kazuo Awai

**Affiliations:** 1 Department of Diagnostic Radiology, Graduate School of Biomedical and Health Sciences, Hiroshima University, 1-2-3 Kasumi, Minami-ku, Hiroshima, 734-8551 Japan; 2 Department of Radiology, Hiroshima University Hospital, 1-2-3 Kasumi, Minami-ku, Hiroshima, 734-8551 Japan; 3 Center for Radiation Disaster Medical Science, Research Institute for Radiation Biology and Medicine, Hiroshima University, 1-2-3 Kasumi, Minami-ku, Hiroshima, 734-8551 Japan; 4 CT Systems Development Department, Canon Medical Systems Corporation, 1385 Shimoishigami, Otawara City, Tochigi 324-8550 Japan

**Keywords:** ^18^F-FDG, PET/CT, Dose reduction, Deep learning-based reconstruction, Image quality

## Abstract

**Background:**

A deep learning-based image reconstruction (DLR) algorithm that can reduce the statistical noise has been developed for PET/CT imaging. It may reduce the administered dose of ^18^F-FDG and minimize radiation exposure while maintaining diagnostic quality. This retrospective study evaluated whether the injected ^18^F-FDG dose could be reduced by applying DLR to PET images. To this aim, we compared the quantitative image quality metrics and the false-positive rate between DLR with a reduced ^18^F-FDG dose and Ordered Subsets Expectation Maximization (OSEM) with a standard dose.

**Results:**

This study included 90 oncology patients who underwent ^18^F-FDG PET/CT. They were divided into 3 groups (30 patients each): group A (^18^F-FDG dose per body weight [BW]: 2.00—2.99 MBq/kg; PET image reconstruction: DLR), group B (3.00–3.99 MBq/kg; DLR), and group C (standard dose group; 4.00—4.99 MBq/kg; OSEM). The evaluation was performed using the signal-to-noise ratio (SNR), target-to-background ratio (TBR), and false-positive rate. DLR yielded significantly higher SNRs in groups A and B than group C (*p* < 0.001). There was no significant difference in the TBR between groups A and C, and between groups B and C (*p* = 0.983 and 0.605, respectively). In group B, more than 80% of patients weighing less than 75 kg had at most one false positive result. In contrast, in group B patients weighing 75 kg or more, as well as in group A, less than 80% of patients had at most one false-positives.

**Conclusions:**

Our findings suggest that the injected ^18^F-FDG dose can be reduced to 3.0 MBq/kg in patients weighing less than 75 kg by applying DLR. Compared to the recommended dose in the European Association of Nuclear Medicine (EANM) guidelines for 90 s per bed position (4.7 MBq/kg), this represents a dose reduction of 36%. Further optimization of DLR algorithms is required to maintain comparable diagnostic accuracy in patients weighing 75 kg or more.

## Introduction

Positron emission tomography combined with computed tomography (PET/CT) using ^18^F-fluorodeoxyglucose (FDG) is a standard imaging modality in oncology for cancer detection and staging, treatment response assessment, and radiotherapy planning [1–3]. However, radiation exposure to patients and healthcare professionals remains a significant concern [4, 5]. As radiation exposure from PET/CT may increase the risk of secondary malignancies and other long-term health effects [6–8], particularly in patients undergoing multiple PET/CT scans for treatment monitoring, reducing the injected dose per scan is an important goal [9, 10].

A deep learning-based image reconstruction (DLR) algorithm has been developed for PET/CT imaging. Compared to conventional methods such as Ordered Subsets Expectation Maximization (OSEM) with Time of Flight (TOF) technology, DLR has demonstrated enhanced noise reduction capabilities, particularly in low-dose imaging scenarios [11–13]. It may help to reduce the administered dose of ^18^F-FDG and minimize radiation exposure while maintaining diagnostic quality. This may contribute to the optimization of nuclear medicine practice [11, 14]. Previous studies [13, 15–17] have evaluated the potential for reducing the ^18^F-FDG dose through simulated images with changes in acquisition time. However, in clinical settings, the effect of body weight (BW) and ^18^F-FDG injected dose on the image quality of DLR cannot be fully accounted for by only considering the acquisition time. To our knowledge, no studies have compared patient groups with different ^18^F-FDG dose per BW to investigate the potential for dose reduction in DLR images. We retrospectively investigated the clinical feasibility of reducing the ^18^F-FDG dose by comparing image quality and false-positive rates between DLR with a reduced ^18^F-FDG dose and OSEM with a standard dose in three patient groups with varying ^18^F-FDG dose per BW.

## Materials and methods

### Patient population

This retrospective study was conducted in accordance with the principles of the Declaration of Helsinki. It was approved by the Ethics Committee for Epidemiology of our institute; the requirement for prior informed consent was waived. The study is in accordance with relevant guidelines and regulations.

Our study included 90 oncology patients who underwent ^18^F-FDG PET/CT scans between March 2023 and March 2024; contrast-enhanced CT or MRI confirmed that none had hepatic tumors. The patient characteristics and primary tumor sites are presented in Tables 1 and 2. We perform PET/CT scans using ^18^F-FDG provided by commercial suppliers. The radiation dose is 185 MBq at 10:00-, 12:30-, and 15:30 h. We administer the entire single dose of ^18^F-FDG intravenously at the scheduled time, and we do not adjust the administered dose based on the time of administration or the patient’s BW. Consequently, the radioactivity of the administered ^18^F-FDG dose varied among the patients; per BW it ranged from 2.00—4.99 MBq/kg.

**Table 1:** Patient characteristics

Patient Group	Group A	Group B	Group C
Patient number	30	30	30
Male/Female	21/9	16/14	16/14
Age^†^	60 [33, 88]	67 [21, 92]	66 [26, 84]
BW (kg) ^*,†^	74 [49,119]	60 [47, 88]	62 [39, 83]
ID (MBq) ^*,†^	188 [135, 265]	215 [162, 338]	295 [175, 370]
ID/BW (MBq/kg) ^*,†^	2.48 [2.20, 2.89]	3.56 [3.02, 3.99]	4.51 [4.00, 4.98]

^*^BW: body weight, ID: Injection dose of ^18^F-FDG

^†^Age, body weight, injected dose, injected dose, and injected dose per body weight are shown as median and range

**Table 2:** Primary tumor sites in each group

Primary site of tumor	Group A	Group B	Group C
Lung	7	10	4
Breast	2	2	3
Esophagus	1	2	3
Stomach	2	0	2
Large intestine	0	3	4
Kidney	1	0	1
Male reproductive organs	0	1	1
Female reproductive organs	4	4	5
Malignant lymphoma	4	6	3
Other	9	2	4

The 90 patients were divided into 3 equal groups based on the injected ^18^F-FDG dose per BW. Group A received 2.00—2.99 MBq/kg; their PET images were generated using DLR. Group B was injected with 3.00–3.99 MBq/kg; their PET images were also subjected to DLR. In group C (standard dose group) we delivered 4.00—4.99 MBq/kg; their PET images were generated using OSEM.

All patients fasted for at least 5 h prior to the PET/CT scans; finger-prick sampling confirmed that their blood glucose levels were ≤ 150 mg/dL. We confirmed that no patients had liver-occupying lesions, i.e. liver tumors or liver cysts and that there were no artifacts due to misregistration between the CT and PET images.

### PET/CT acquisition and image reconstruction

The patients received an ^18^F-FDG injection; PET/CT scans were performed approximately 60 min later. Images were acquired from the vertex to the mid-thigh using a PET/CT system (Cartesion Prime, Canon Medical Systems, Japan). Before PET imaging, a CT scan was acquired with a fixed tube voltage of 120 kV and an automatic mAs modulation technique to enable attenuation correction and anatomical localization. Subsequently, PET acquisition was performed in three-dimensional (3D) list mode with a 40–50% overlap, and all images were reconstructed from the list mode with a uniform acquisition time of 90 s per bed position. The OSEM algorithm was reconstructed with 2 iterations, 12 subsets, a Gaussian filter with 4-mm full width at half maximum; TOF and point-spread function (PSF) corrections were applied.

The DLR was a reconstruction algorithm that is commercially available and installed in our PET/CT scanner (Advanced Intelligent Clear-IQ Engine—integrated: AiCE-i, Canon Medical Systems). Technical details of the DLR we used are described elsewhere [11, 14].

### Quantitative analysis

Quantitative analysis was on a workstation (Advantage Workstation, GE Healthcare, Japan); the signal-to-noise- and the target-to-background ratios were calculated (SNR, TBR) and served as the quantitative indices [18–20]. To calculate the SNR we first identified the slice with the largest hepatic cross-sectional area on horizontal cross-sectional CT images. Then we placed a 50-mm diameter region of interest (ROI) on the horizontal PET slice that corresponded with the CT slice level and on the preceding and subsequent PET slice and measured the mean standardized uptake value (SUV_mean_) and its standard deviation (SD). The ROI was placed in the liver away from the liver margin to avoid partial volume effects. The SNR of each ROI was calculated by dividing the SUV_mean_ by the SD. Finally, we averaged the SNR for the 3 slices to obtain the SNR for that patient.

The TBR was calculated by dividing the maximum standardized uptake value (SUVmax) of the target by the mean standardized uptake value (SUVmean) of the background. The SUVmax of the target was measured by placing a ROI that encompassed the entire lesion. The target was defined as the primary tumor showing FDG uptake on PET images, or if no uptake was observed in the primary tumor, as an FDG-avid lymph node or hematogenous metastasis. In patients with multiple lesions, the one with the highest uptake was selected for measurement. The mean target ROI diameter was 26 mm. The background SUVmean was obtained by placing a 10-mm diameter ROI within the descending aorta, avoiding the aortic wall and calcifications. The long diameter of the target lesion was measured on the maximum cross-sectional area using CT portion of PET/CT.

### Visual analysis of false-positives

In all patients, two radiologists with 21 and 9 years of experience, respectively, in interpreting PET/CT images were assigned to visually assess the presence or absence of false-positive hepatic lesions on PET images of randomly assigned patients. The patient histories and the reconstruction algorithms applied were unknown to the readers. The absence of hepatic lesions was confirmed by contrast-enhanced CT and/or MRI. The liver was selected because it is the only organ in which FDG uptake is relatively uniform [18]. The reference images for determining false-positives were OSEM images acquired with a median acquisition time of 162 s (range: 90—438 s). In all patients, false-positives were defined as accumulations that were significantly more accumulated than the corresponding reference images of the same patient. The number of false-positives was independently assessed by 2 observers; final decisions were by consensus. Based on the consensus number of false-positives, images with one or no false-positives were recorded as diagnostically acceptable, images with two or more false-positives as unacceptable.

Each group was divided into patients weighing less than 75 kg and patients whose BW was 75 kg or more and the number of diagnostically acceptable and unacceptable images was recorded. When the rate of acceptable images in each BW group was 80% or higher, images of the entire group were considered to be diagnostically acceptable.

### Statistical analysis

The SNR and TBR obtained by quantitative analysis of the 3 groups were evaluated using the Kruskal–Wallis test followed by Steel’s test for *post-hoc* comparisons. For the Steel test, group C served as the standard; comparison was made between groups A and C (standard dose), and B and C.

In the visual assessment of false-positives, the kappa test was used to evaluate the inter-reader agreement.

Statistical significance was defined as a p-value of less than 0.05. All statistical tests were conducted using EZR (Saitama Medical Center, Jichi Medical University, Saitama, Japan).

## Results

### Quantitative analysis of image quality

**Fig. 1:**
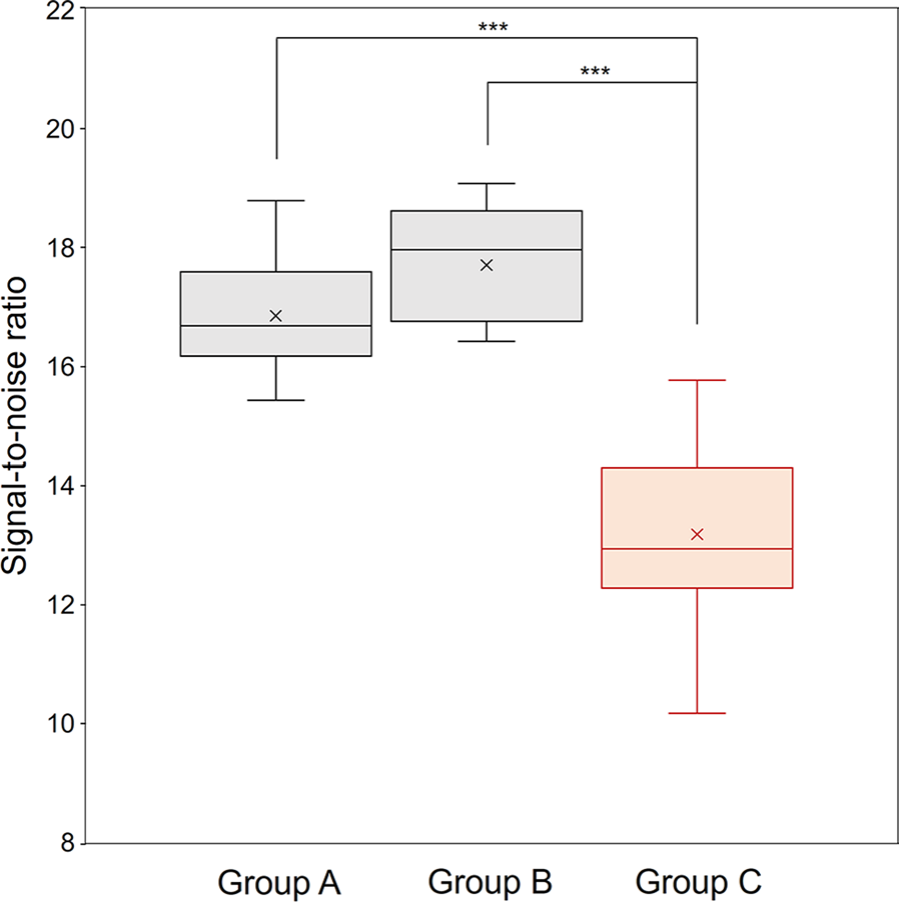
Comparison of the SNR between groups A (2.00–2.99 MBq/kg) and C (standard dose, 4.00—4.99 MBq/kg); and groups B (3.00–3.99 MBq/kg) and C. Groups A and B showed a substantial improvement in SNR compared to group C (*p* < 0.001)

Figure 1 compares the SNR between groups A and C (standard dose), and groups B and C. It was significantly higher in groups A and B than in group C (p < 0.001).

**Fig. 2:**
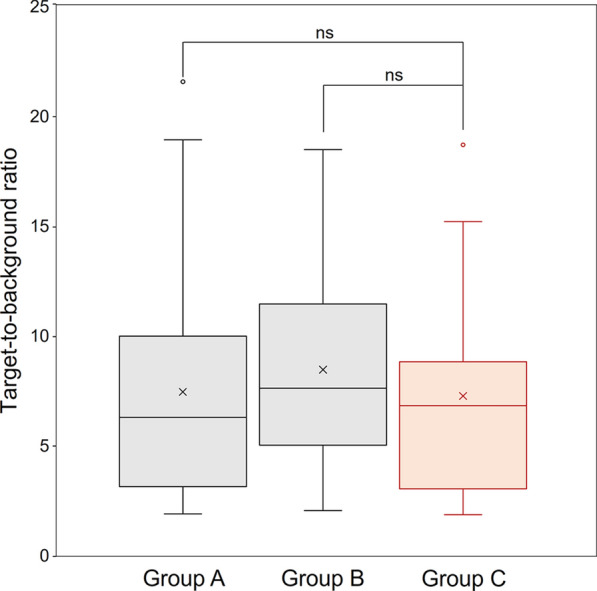
Comparison of the TBR between groups A (2.00—2.99 MBq/kg) and C (standard dose, 4.00—4.99 MBq/kg); and groups B (3.00—3.99 MBq/kg) and C. No significant difference in TBR was observed between group C and groups A and B. ns: no significant difference

FDG uptake in primary tumors, lymph node metastases, or hematogenous metastases was confirmed by PET/CT in 22 patients in group A, 18 in group B, and 20 in group C, and a total of 60 lesions were used for TBR evaluation. In contrast, no such abnormal uptake was observed in 8 patients in group A, 12 in group B, and 10 in group C. Figure 2 compares the TBR between groups A and C (standard dose), and groups B and C. There was no statistically significant difference in the TBR of these groups (p = 0.983 and 0.605, respectively) although the median TBR in group B was 12% higher than in group C. The long diameter of the target lesions ranged from 7 to 81 mm.

### Visual analysis of false positives

Table 3 shows the median and the range of false-positive lesions recorded by the two readers and the kappa coefficients for each patient group.

**Table 3:** False positives in each group and reader with inter-reader kappa coefficients

	Group A	Group B	Group C (standard dose)
Number of false positives for observer 1^*^	2 [0, 5]	0 [0, 4]	0 [0, 1]
Number of false positives for observer 2^*^	2 [0, 7]	0 [0, 5]	0 [0, 1]
Kappa coefficient between observers 1 and 2	0.556	0.730	1.00

^*^Number indicates median number of false positives and numbers in parenthesis indicate range of false positive number

In group A, the median number of false-positives was 2, in groups B and C (standard dose) it was 0. The kappa coefficients between the readers were 0.556, 0.730, and 1.000 for groups A, B, and C, respectively.

Table 4 lists the number of patients with false-positive lesions in each BW group. Each group was further subdivided into patients weighing less than 75 kg and those weighing 75 kg or more. In group B, more than 80% of patients weighing less than 75 kg had at most one false-positive lesions. In contrast, in group B patients weighing 75 kg or more, as well as in group A, less than 80% of patients had at most one false-positive lesions. In group C (standard dose), there was one patient with one false positive result.

**Table 4:** The number of false positives classified by each group and by individual number

Number of FP^*^	Group								
	A			B			C (standard dose)		
	FP = 0	FP = 1	2 ≤ FP	FP = 0	FP = 1	2 ≤ FP	FP = 0	FP = 1	2 ≤ FP
Number of patients weighing less than 75 kg	5/16 (31%)	6/16 (38%)	5/16 (31%)	16/25 (64%)	5/25 (20%)	4/25 (16%)	26/26 (100%)	0/26 (0%)	0/26 (0%)
Number of patients weighing 75 kg or more	0/14 (0%)	3/14 (21%)	11/14 (79%)	0/5 (0%)	0/5 (0%)	5/5 (100%)	3/4 (75%)	1/4 (25%)	0/4 (0%)
Overall	5/30 (17%)	9/30 (30%)	16/30 (53%)	16/30 (53%)	5/30 (17%)	9/30 (30%)	29/30 (97%)	1/30 (3%)	0/30 (0%)

^*^FP: False Positive

**Fig. 3:**
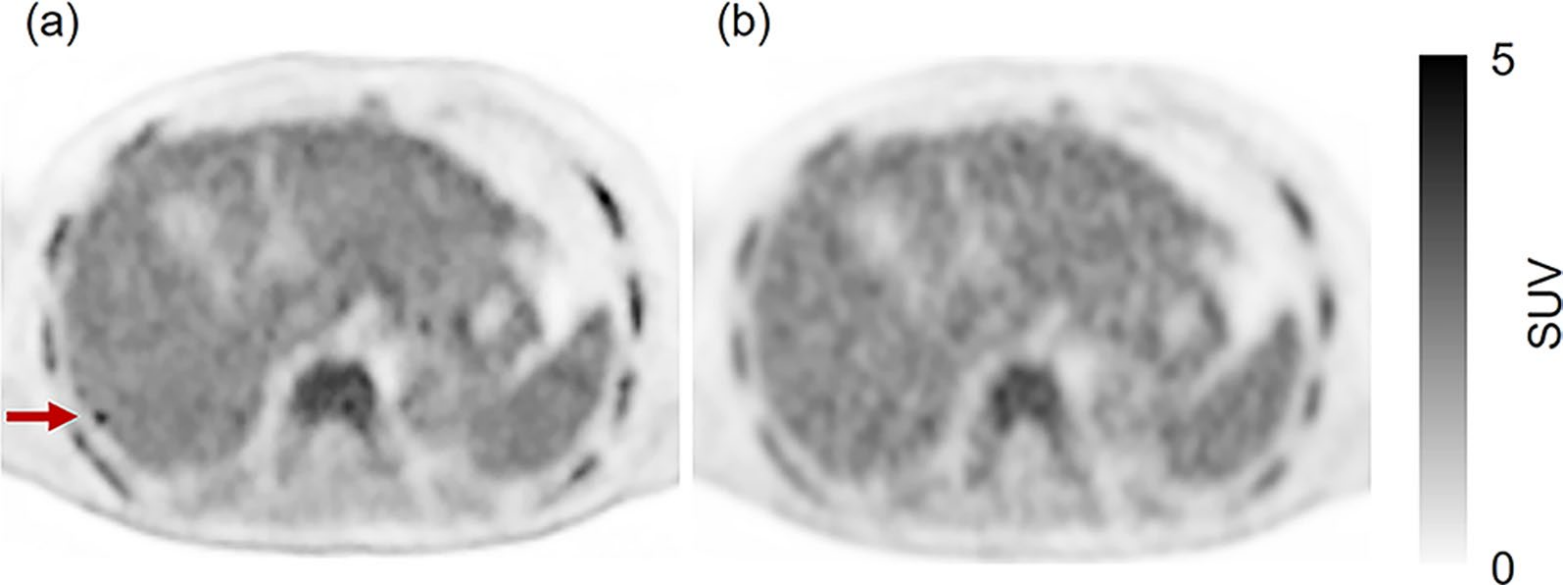
A 52- year-old male in group A. His body weight was 74.0 kg. He was injected with 169 MBq of ^18^F-FDG (2.28 MBq/kg). One false-positive lesion was recorded. **a**: PET image reconstructed with DLR of data acquired in 90 s. **b**: PET image reconstructed with OSEM using data acquired with the maximum acquisition time (248 s). The image is a reference image. Although there was an abnormal uptake of ^18^F-FDG on the DLR image (**a**, arrow), there was no abnormal uptake on the OSEM reference image (**b**). The abnormal uptake on the DLR image was confirmed as a false-positive

**Fig. 4:**
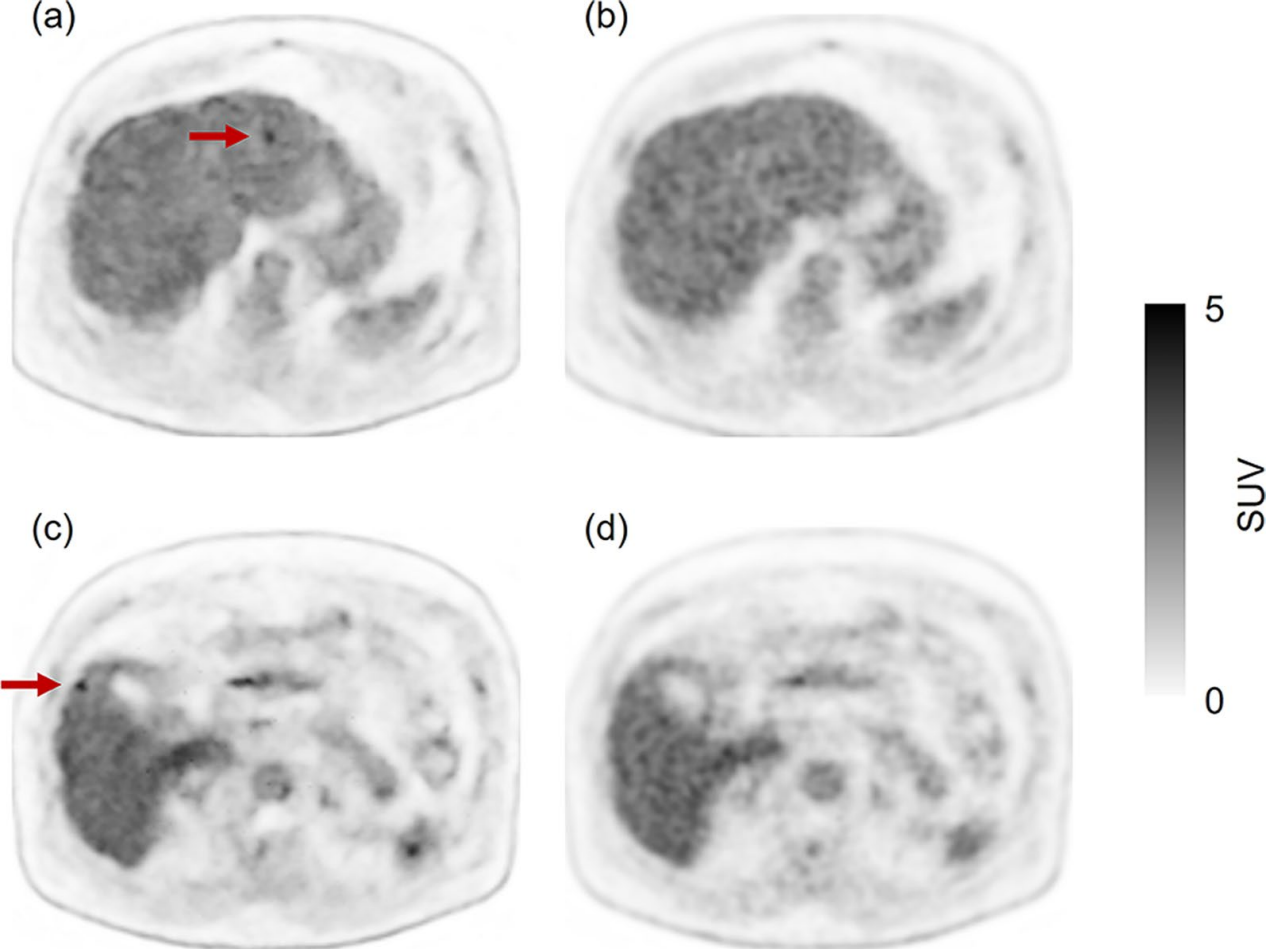
A 59-year-old male in group A. His body weight was 92.7 kg. He was injected with 214 MBq of ^18^F-FDG (2.31 MBq/kg). Five false-positive lesions were recorded. **a** and **c**: PET images reconstructed with DLR from data acquired in 90 s. **b** and **d**: PET images reconstructed with OSEM using data acquired in the maximum acquisition time (320 s). Images **b** and **d** are the reference images

**Fig. 5:**
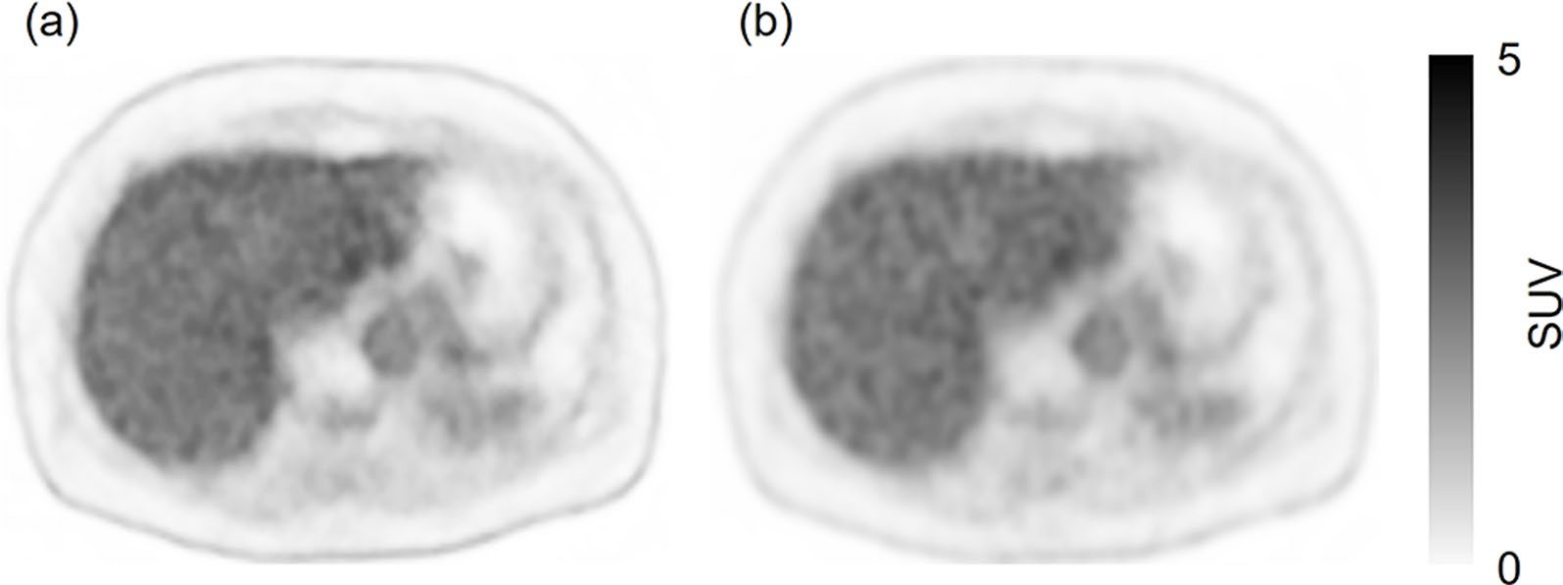
A 61-year-old female in group B. Her body weight was 65.9 kg. She was injected with 229 MBq of ^18^F-FDG (3.47 MBq/kg). There were no false-positive lesions. **a**: PET image reconstructed with DLR from data acquired in 90 s. **b**: PET image reconstructed with OSEM using data acquired in the maximum acquisition time (169 s). **b** is the reference image

**Fig. 6:**
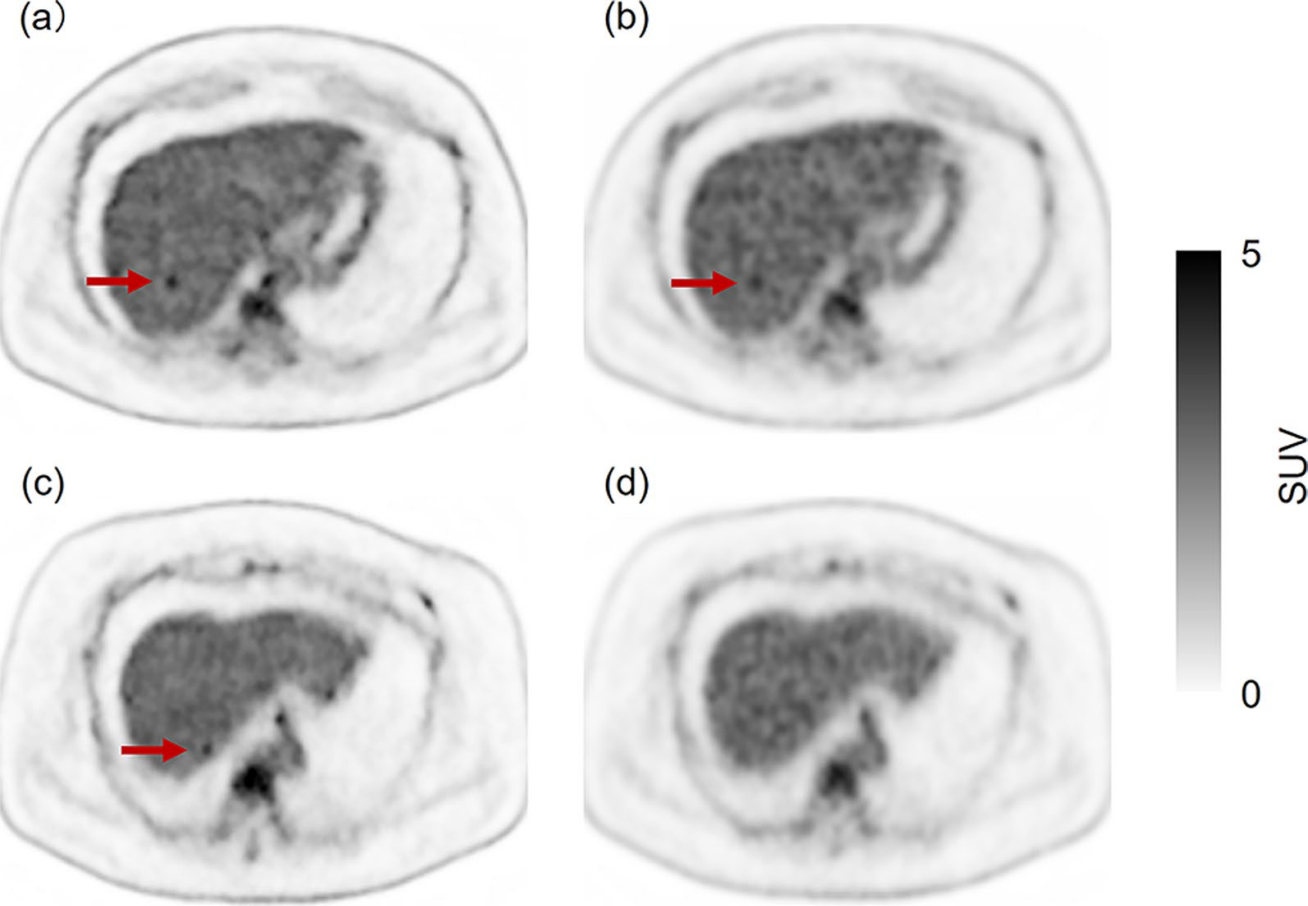
A 47 -year- old female in group B. Her body weight was 87.6 kg. She was injected with 338 MBq of ^18^F-FDG (3.86 MBq/kg). Four false-positive lesions were recorded. **a** and **c**: PET images reconstructed with DLR from data acquired in 90 s. **b** and **d**: PET images reconstructed with OSEM using data acquired in the maximum acquisition time (222 s). Images **b** and **d** are reference images. Although there was an uptake of ^18^F-FDG on the DLR image (**a**), the same uptake was observed on the OSEM reference image (**b**). As neither CT nor MRI confirmed hepatic lesions, the uptake on DLR and OSEM images was confirmed as false-positive. Although there was an abnormal uptake of ^18^F-FDG on the DLR image (**c**), no abnormal uptake is seen on the OSEM reference image (**d**). The uptake on the DLR image was confirmed as a false positive

Representative cases are shown in Figs. 3, 4, 5, and 6.

## Discussion

In this study, we investigated the feasibility of dose reduction using DLR by performing an intergroup comparison of 90 patients with different ^18^F-FDG dose per BW under a fixed acquisition time. Although the ^18^F-FDG dose per BW was lower in groups A and B than in group C (standard dose), their SNR was statistically higher than in group C; their TBR was equivalent to that of group C. Based on quantitative metrics, with DLR the ^18^F-FDG dose could potentially be reduced to 2.0 MBq/kg BW. On the other hand, when evaluating false-positive lesions in the liver, in Group A, more than half (53%) of the patients had two or more false-positive lesions. In contrast, in Group B, two or more false-positive lesions were observed in all patients with a body weight of 75 kg or more, but in patients weighing less than 75 kg, 84% had one or fewer false-positive lesions, which was within the acceptable diagnostic range. Based on our results with DLR, we believe that the ^18^F-FDG dose can be reduced to 3.00 MBq/kg BW in patients weighing less than 75 kg, which is a 36% reduction compared to the dose of 4.7 MBq/kg BW for 90 s per bed position recommended by the EANM guidelines [1]. We used the DLR(AiCE-i) developed by Canon Medical Systems. Tsuchiya et al. [11] reported that DLR(AiCE-i) showed significantly higher scores in tumor visualization, overall image quality, and noise reduction compared to 3D TOF-OSEM filtered with a Gaussian filter. However, they did not investigate whether DLR(AiCE-i) could enable ^18^F-FDG dose reduction. Our study demonstrated that dose reduction is achievable with DLR(AiCE-i) using data based on the actual injected ^18^F-FDG dose per BW.

PET/CT with DLR developed by other manufacturers has also been reported to allow for a dose reduction [13, 15–17]. Xing et al.[13] reported that PET/CT using DLR (HYPER DLR™) in 52 patients maintained image quality comparable to reference images, even with a 25% reduction in FDG dose. According to Ciborowski et al. [17], PET/CT using DLR (SubtlePET™) in 37 patients showed that image quality was not compromised even when the FDG dose was reduced by 33%. However, these studies were conducted using simulated images with varying acquisition times generated from list-mode data. In contrast, the present study focused on dose reduction using DLR by performing an intergroup comparison of 90 patients who received different ^18^F-FDG dose per BW.

Xing et al.[13] also found that DLR was effective in patients with a high body mass index. However, in our study, the number of false-positive lesions increased with the patient’s BW. The rate of patients with two or more false-positive lesions was 79% in group A (^18^F-FDG dose: 2.00–2.99 MBq/kg) and 100% in group B (3.00–3.99 MBq/kg) among patients with a BW of 75 kg or more. The training data for our DLR (AiCE-i) are not publicly available, and probably included only few patients weighing 75 kg or more. In the future, it may be possible to reduce the number of false positives in patients with a BW of 75 kg or more by increasing the number of PET/CT data used as training data for the DLR.

Previous studies[17, 21] have suggested the possibility of dose reduction based on the assumption that acquisition time and injected dose have an equivalent effect on image quality. Based on the same premise, the results of the present study may also suggest the potential for shortening acquisition time. However, since the relationship between acquisition time and dose may not be consistent depending on patient body habitus and scanner sensitivity[18, 22, 23], further investigation is warranted to evaluate the feasibility of acquisition time reduction.

Although the ^18^F-FDG dose per body weight was lower in groups A and B than in group C (standard dose), the SNR was statistically significantly higher in groups A and B. The noise reduction process of DLR (AiCE-i) involves training the network by using noisy data as input and high-quality data as the learning target, thereby enabling the reconstruction of clean images from noise-contaminated ones. As a result, DLR selectively removes noise more effectively than conventional methods using Gaussian filters[11]. This explains why groups A and B, despite receiving a lower FDG dose per body weight, demonstrated higher SNR than group C (OSEM with standard dose). However, in DLR, SNR the conventional metric for evaluating noise reduction may not necessarily reflect the presence or absence of clinically meaningful features [24, 25]. Therefore, in this study, we also performed visual assessment of false positives to explore the potential for clinically applicable dose reduction.

In this study, we considered a case to be diagnostically acceptable if the number of false-positive lesions was 1 or zero. This is because even with OSEM images of patients who have received the standard dose of ^18^F-FDG can sometimes show a false positive. Chua et al. [26] who investigated the diagnostic performance of ^18^F-FDG PET/CT (^18^F-FDG dose: 370 MBq) in patients with liver metastases reported that there were 2 false-positive lesions in 131 patients. Although the number of false-positive results per patient is not specified in this article, it is estimated to be 1 based on the contents of the article, so it is estimated that about 1 false-positive result per patient may occur even with OSEM. Beiderwellen et al. [27] compared the diagnostic performance of PET/MRI and PET/CT in ^18^F-FDG-injected patients for the detection of liver metastases; they found that PET/CT revealed one false-positive lesion in one of 32 patients. In our study, there was also one patient in group C (OSEM image reconstruction) who had a false-positive lesion.

This study has several limitations that should be acknowledged. First, the 3 patient groups differed in the BW; it was difficult to address this issue due to the retrospective nature of the study. We are planning a prospective study in the future. Second, the observers were not informed whether the images were reconstructed with DLR or OSEM in their visual assessment of false positives. However, as both were experienced radiologists with experience in interpreting PET/CT scans, the reconstruction method may have been obvious to them. Third, the PET scan overlap (40–50%) varied between patients, which may have affected the SNR and TBR; however, in clinical practice, it is often difficult to standardize the overlap across all patients. McKeown et al.[28] reported that the effect of different overlap on image quality was minimal. In the present study, intergroup comparisons were performed while accounting for the variability in overlap, thereby allowing for the evaluation of SNR and TBR under realistic clinical conditions. Finally, we applied the DLR algorithm to ^18^F-FDG PET/CT images. We do not know whether similar results would have been obtained if we had used other tracers. Separate studies with different tracers may be needed to obtain comparable results.

## Conclusion

To our knowledge, this study is the first to evaluate how much the ^18^F-FDG dose can be reduced by applying DLR using clinical data with different ^18^F-FDG dose per BW. DLR yielded diagnostically acceptable images when the ^18^F-FDG dose was 3.0 MBq/kg in patients weighing less than 75 kg. This is a 36% dose reduction from the EANM guideline recommended dose for 90 s per bed position (4.7 MBq/kg).

Advances in DLR technology are needed to achieve high quality images in patients weighing 75 kg or more.

## Data Availability

The datasets generated during and/or analyzed during the current study are available from the corresponding author on reasonable request.

## Abbreviations

3D: Three-dimensional
BW: Body weight
DLR: Deep learning-based image reconstruction
EANM: European association of nuclear medicine
FDG: Fluorodeoxyglucose
OSEM: Ordered subsets expectation maximization
PET/CT: Positron emission tomography combined with computed tomography
PSF: Point-spread function
ROI: Region of interest
SD: Standard deviation
SNR: Signal-to-noise ratio
SUV: Standardized uptake value
TBR: Target-to-background ratio
TOF: Time of flight

## References

[1] Boellaard R, Delgado-Bolton R, Oyen WJ, Giammarile F, Tatsch K, Eschner W, et al. FDG PET/CT: EANM procedure guidelines for tumour imaging: version 2.0. Eur J Nucl Med Mol Imaging. 2015;42(2):328–54. 25452219 10.1007/s00259-014-2961-x PMC4315529

[2] Fletcher JW, Djulbegovic B, Soares HP, Siegel BA, Lowe VJ, Lyman GH, et al. Recommendations on the use of 18F-FDG PET in oncology. J Nucl Med. 2008;49(3):480–508. 18287273 10.2967/jnumed.107.047787

[3] Weber WA. Use of PET for monitoring cancer therapy and for predicting outcome. J Nucl Med. 2005;46(6):983–95. 15937310

[4] Nievelstein RA, Quarles van Ufford HM, Kwee TC, Bierings MB, Ludwig I, Beek FJ, et al. Radiation exposure and mortality risk from CT and PET imaging of patients with malignant lymphoma. Eur Radiol. 2012;22(9):1946–54. 22538627 10.1007/s00330-012-2447-9 PMC3411290

[5] Erdemir RU, Abuzaid MM, Cavli B, Tekin HO, Elshami W. Assessment of extremity dose for medical staff involved in positron emission tomography/computed tomography imaging: retrospective study. Medicine. 2023;102(43): e35501. 37904454 10.1097/MD.0000000000035501 PMC10615540

[6] Wen JC, Sai V, Straatsma BR, McCannel TA. Radiation-related cancer risk associated with surveillance imaging for metastasis from choroidal melanoma. JAMA Ophthalmol. 2013;131(1):56–61. 23307209 10.1001/jamaophthalmol.2013.564

[7] Huang B, Law MW, Khong PL. Whole-body PET/CT scanning: estimation of radiation dose and cancer risk. Radiology. 2009;251(1):166–74. 19251940 10.1148/radiol.2511081300

[8] Prasad A, Visweswaran S, Kanagaraj K, Raavi V, Arunan M, Venkatachalapathy E, et al. (18)F-FDG PET/CT scanning: Biological effects on patients: Entrance surface dose, DNA damage, and chromosome aberrations in lymphocytes. Mutat Res Genet Toxicol Environ Mutagen. 2019;838:59–66. 30678829 10.1016/j.mrgentox.2018.12.010

[9] Iovoli AJ, Farrugia MK, Ma SJ, Chan JM, Markiewicz MR, McSpadden R, et al. Role of repeat PET/CT imaging in head and neck cancer following initial incomplete PET/CT response to chemoradiation. Cancers. 2021;13(6):1461. 33806792 10.3390/cancers13061461 PMC8004876

[10] Castello A, Rossi S, Lopci E. 18F-FDG PET/CT in restaging and evaluation of response to therapy in lung cancer: state of the art. Curr Radiopharm. 2020;13(3):228–37. 31886757 10.2174/1874471013666191230144821 PMC8493792

[11] Tsuchiya J, Yokoyama K, Yamagiwa K, Watanabe R, Kimura K, Kishino M, et al. Deep learning-based image quality improvement of (18)F-fluorodeoxyglucose positron emission tomography: a retrospective observational study. EJNMMI Phys. 2021;8(1):31. 33765233 10.1186/s40658-021-00377-4 PMC7994470

[12] Bonardel G, Dupont A, Decazes P, Queneau M, Modzelewski R, Coulot J, et al. Clinical and phantom validation of a deep learning based denoising algorithm for F-18-FDG PET images from lower detection counting in comparison with the standard acquisition. EJNMMI Phys. 2022;9(1):36. 35543894 10.1186/s40658-022-00465-z PMC9095795

[13] Xing Y, Qiao W, Wang T, Wang Y, Li C, Lv Y, et al. Deep learning-assisted PET imaging achieves fast scan/low-dose examination. EJNMMI Phys. 2022;9(1):7. 35122172 10.1186/s40658-022-00431-9 PMC8816983

[14] Yamagiwa K, Tsuchiya J, Yokoyama K, Watanabe R, Kimura K, Kishino M, et al. Enhancement of (18)F-Fluorodeoxyglucose PET image quality by deep-learning-based image reconstruction using advanced intelligent clear-IQ engine in semiconductor-based PET/CT scanners. Diagnostics. 2022;12(10):2500. 36292189 10.3390/diagnostics12102500 PMC9599974

[15] Yan L, Wang Z, Li D, Wang Y, Yang G, Zhao Y, et al. Low (18)F-fluorodeoxyglucose dose positron emission tomography assisted by a deep-learning image-denoising technique in patients with lymphoma. Quant Imag Med Surg. 2024;14(1):111–22. 10.21037/qims-23-817 PMC10784027 38223079

[16] Katsari K, Penna D, Arena V, Polverari G, Ianniello A, Italiano D, et al. Artificial intelligence for reduced dose 18F-FDG PET examinations: a real-world deployment through a standardized framework and business case assessment. EJNMMI Phys. 2021;8(1):25. 33687602 10.1186/s40658-021-00374-7 PMC7943690

[17] Ciborowski K, Gramek-Jedwabna A, Golab M, Miechowicz I, Szczurek J, Ruchala M, et al. Performance of a deep learning enhancement method applied to PET images acquired with a reduced acquisition time. Nucl Med Rev Cent East Eur. 2023;26:116–22. 37786943 10.5603/nmr.94482

[18] de Groot EH, Post N, Boellaard R, Wagenaar NR, Willemsen AT, van Dalen JA. Optimized dose regimen for whole-body FDG-PET imaging. EJNMMI Res. 2013;3(1):63. 23938036 10.1186/2191-219X-3-63 PMC3751692

[19] Cheng Z, Chen L, Wang X, Wang Y, Zhao M, Zan K, et al. Role of breath-hold lung PET in stage IA pulmonary adenocarcinoma. Insights Imaging. 2023;14(1):100. 37227573 10.1186/s13244-023-01446-1 PMC10212906

[20] Zhang P, Chen W, Zhao K, Qiu X, Li T, Zhu X, et al. Tumor to liver maximum standardized uptake value ratio of FDG-PET/CT parameters predicts tumor treatment response and survival of stage III non-small cell lung cancer. BMC Med Imag. 2023;23(1):107. 10.1186/s12880-023-01067-6 PMC10428530 37582701

[21] Ghafari A, Sheikhzadeh P, Seyyedi N, Abbasi M, Farzenefar S, Yousefirizi F, et al. Generation of(18)F-FDG PET standard scan images from short scans using cycle-consistent generative adversarial network. Phys Med Biol. 2022;67(21):215005. 10.1088/1361-6560/ac950a 36162408

[22] Sagara H, Inoue K, Yaku H, Ohsawa A, Someya T, Yanagisawa K, et al. Optimization of injection dose in (18)F-FDG PET/CT based on the 2020 national diagnostic reference levels for nuclear medicine in Japan. Ann Nucl Med. 2021;35(11):1177–86. 34287782 10.1007/s12149-021-01656-x PMC8494693

[23] Masuda Y, Kondo C, Matsuo Y, Uetani M, Kusakabe K. Comparison of imaging protocols for 18F-FDG PET/CT in overweight patients: optimizing scan duration versus administered dose. J Nucl Med. 2009;50(6):844–8. 19443586 10.2967/jnumed.108.060590

[24] Bradshaw TJ, Boellaard R, Dutta J, Jha AK, Jacobs P, Li Q, et al. Nuclear Medicine and Artificial Intelligence: Best Practices for Algorithm Development. J Nucl Med. 2022;63(4):500–10. 34740952 10.2967/jnumed.121.262567 PMC10949110

[25] Balaji V, Song TA, Malekzadeh M, Heidari P, Dutta J. Artificial intelligence for PET and SPECT image enhancement. J Nucl Med. 2024;65(1):4–12. 37945384 10.2967/jnumed.122.265000 PMC10755520

[26] Chua SC, Groves AM, Kayani I, Menezes L, Gacinovic S, Du Y, et al. The impact of 18F-FDG PET/CT in patients with liver metastases. Eur J Nucl Med Mol Imaging. 2007;34(12):1906–14. 17713766 10.1007/s00259-007-0518-y

[27] Beiderwellen K, Geraldo L, Ruhlmann V, Heusch P, Gomez B, Nensa F, et al. Accuracy of [18F]FDG PET/MRI for the detection of liver metastases. PLoS ONE. 2015;10(9): e0137285. 26335246 10.1371/journal.pone.0137285 PMC4559465

[28] McKeown C, Gillen G, Dempsey MF, Findlay C. Influence of slice overlap on positron emission tomography image quality. Phys Med Biol. 2016;61(3):1259–77. 26788967 10.1088/0031-9155/61/3/1259

